# Dead in the Night: Sleep-Wake and Time-Of-Day Influences on Sudden Unexpected Death in Epilepsy

**DOI:** 10.3389/fneur.2018.01079

**Published:** 2018-12-11

**Authors:** Benton S. Purnell, Roland D. Thijs, Gordon F. Buchanan

**Affiliations:** ^1^Department of Neurology, Carver College of Medicine, University of Iowa, Iowa City, IA, United States; ^2^Neuroscience Program, University of Iowa, Iowa City, IA, United States; ^3^Iowa Neuroscience Institute, Carver College of Medicine, University of Iowa, Iowa City, IA, United States; ^4^Stichting Epilepsie Instellingen Nederland (SEIN), Heemstede, Netherlands; ^5^NIHR University College London Hospitals Biomedical Research Centre, UCL Institute of Neurology, London, United Kingdom; ^6^Department of Neurology, LUMC Leiden University Medical Center, Leiden, Netherlands

**Keywords:** SUDEP, sleep, epilepsy, circadian rhythms, breathing

## Abstract

Sudden unexpected death in epilepsy (SUDEP) is the leading cause of epilepsy-related death in patients with refractory epilepsy. Convergent lines of evidence suggest that SUDEP occurs due to seizure induced perturbation of respiratory, cardiac, and electrocerebral function as well as potential predisposing factors. It is consistently observed that SUDEP happens more during the night and the early hours of the morning. The aim of this review is to discuss evidence from patient cases, clinical studies, and animal research which is pertinent to the nocturnality of SUDEP. There are a number of factors which might contribute to the nighttime predilection of SUDEP. These factors fall into four categories: influences of (1) being unwitnessed, (2) lying prone in bed, (3) sleep-wake state, and (4) circadian rhythms. During the night, seizures are more likely to be unwitnessed; therefore, it is less likely that another person would be able to administer a lifesaving intervention. Patients are more likely to be prone on a bed following a nocturnal seizure. Being prone in the accouterments of a bed during the postictal period might impair breathing and increase SUDEP risk. Sleep typically happens at night and seizures which emerge from sleep might be more dangerous. Lastly, there are circadian changes to physiology during the night which might facilitate SUDEP. These possible explanations for the nocturnality of SUDEP are not mutually exclusive. The increased rate of SUDEP during the night is likely multifactorial involving both situational factors, such as being without a witness and prone, and physiological changes due to the influence of sleep and circadian rhythms. Understanding the causal elements in the nocturnality of SUDEP may be critical to the development of effective preventive countermeasures.

## Introduction

The leading cause of epilepsy-related death in patients with refractory epilepsy is sudden unexpected death in epilepsy [SUDEP; (1–5)]. Among neurological conditions, SUDEP is second only to stroke in terms of years of potential life lost to disease (4). There are no effective ways to reliably predict or prevent SUDEP (3, 5–8). SUDEP is hypothesized to be the result of predisposing factors in the patient and seizure induced perturbation of respiratory, cardiac, and electrocerebral function (3, 5, 7–9). In cases of SUDEP which have been recorded in an epilepsy monitoring unit, respiratory arrest appears to be the primary cause of death as terminal apnea precedes terminal asystole in each case (10).

It is consistently observed that SUDEP happens more during the night and the early hours of the morning (2, 10–14). Lamberts et al. observed that 62% of SUDEP cases happened between midnight and noon and that 58% of SUDEP cases were sleep-related (12). In SUDEP cases occurring in epilepsy monitoring units, 87.5% of deaths were observed to happen during the night (10). In a meta-analysis of definite, probable, and possible SUDEP, Ali et al. observed that 69.3% of SUDEP cases were presumed to have happened during sleep (13). Furthermore, patients who die of SUDEP are about twice as likely to have nocturnal seizures than those who did not die of SUDEP (12, 14). The increased nocturnal incidence of SUDEP is often attributed to an increased risk of SUDEP during sleep; however, there are a number of factors which might contribute to the nighttime predilection of SUDEP. These factors fall into four categories: influences of (1) being in the absence of a witness, (2) lying prone in bed, (3) sleep-wake state, and (4) circadian rhythms. A consistent issue for determining the cause of the nocturnality of SUDEP is disentangling the potential effect of sleep from the effect of circadian rhythms, not to mention complicating factors such as being without a witness and prone in bed. In humans, sleep typically happens during the night. Consequently, circadian rhythms and homeostatic sleep processes are often considered together; nevertheless, these are distinct processes. Indeed, sleep and circadian rhythmicity alter physiologic processes, such as cardiac and respiratory function, independent of one another (15–20). For a comprehensive meta-analysis of SUDEP cases which consider sleep state or time-of-day as a potential risk factor, please see (13) and (21). The aim of this review is to discuss evidence from patient cases, clinical studies, and animal research which is pertinent to the nocturnality of SUDEP and to consider the implications for clinicians, patients, and the development of preventative strategies. The definition of SUDEP established by Nashef et al. is used for the purposes of this review unless otherwise specified (22).

## Being in the Absence of A Witness

Most SUDEP cases are unwitnessed (12, 23). This suggests that the presence of someone who could intervene after a seizure may be protective against SUDEP (2, 12). Seizures which happen during the hours of the day usually occupied by sleep are more likely to be unwitnessed than those occurring during wakefulness (24, 25). Increasing nocturnal supervision by the use of monitoring devices, regular checks or having someone else sleep in the same room is associated with a decreased risk of SUDEP (2, 6, 14). The mechanism by which the presence of another person might differentiate survival from SUDEP is not clear; however, nursing interventions such as repositioning and supplemental oxygen administration are associated with shorter seizures, a reduction in postictal EEG suppression and improved respiratory function (23, 26).

Given the potential for life saving interventions in the time after a severe seizure, the development and distribution of devices capable of predicting seizures and/or detecting seizures and alerting others holds great promise for reducing the rate of nighttime SUDEP. Accurate seizure forecasting would potentially allow for preventative measures to be taken to reduce the chance that an approaching seizure results in SUDEP. Unfortunately, seizure forecasting has proved quite challenging (27).

Conversely, automated seizure detection devices have the potential to detect convulsive seizures with some degree of reliability (28, 29). While EEG is still the most reliable modality for seizure detection, an EEG apparatus is likely not realistic in the home setting. Additionally, while over-night video monitoring improves the detection of nocturnal seizures in a clinical setting it may not be reasonable to expect someone to monitor patients in this way in the home setting (30). The development of automated seizure detection algorithms which use video data to trigger an alarm in response to seizures have considerable promise for reducing the rate of nocturnal SUDEP (31). Unfortunately, there is a scarcity of long-term home-based data to support the efficacy of nocturnal monitoring and seizure detection devices (32, 33). Furthermore, reliable alarms only have the potential to prevent death if there is another person who is able to quickly intervene in response to the alarm. Lastly, increased monitoring of at risk patients by caregivers or devices is unlikely to be successful in all cases as SUDEP has been known to occur even in the presence of medical professionals after the patient announced “I'm going to have a seizure” (34, 35).

## Lying Prone in Bed

**Figure 1:**
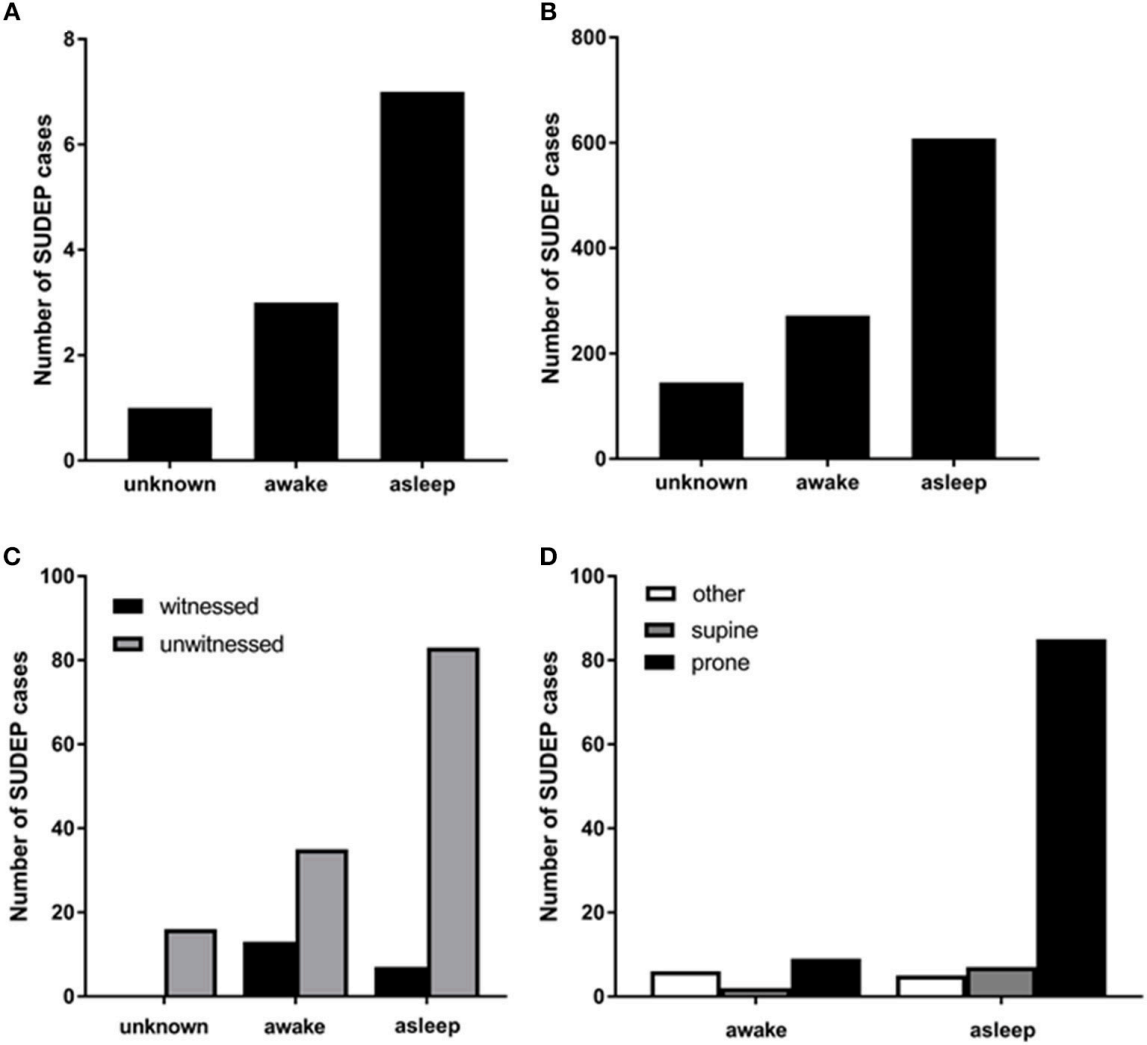
**(A)** Numbers of SUDEP cases in different vigilance states captured by EEG in the mortality in epilepsy monitoring units study (MORTEMUS: redrawn with permission from Ryvlin et al. (10). **(B)** numbers of sleep-related definite, possible, and probable SUDEP cases [redrawn with permission from Ali et al. (13)]; **(C)** numbers of witnessed and unwitnessed SUDEP cases in sleep and wakefulness [redrawn with permission from Lamberts et al. (12)]. **(D)** numbers of definite, possible, and probable SUDEP cases in sleep and wakefulness and in different body positions [redrawn with permission from Ali et al. (13)].

In the majority of SUDEP cases, the victim is found in the prone position regardless of the supposed vigilance state of seizure origin (36–38); however, possible, probable and definite SUDEP cases which are inferred to have happened during sleep are more likely to be found prone than those which are inferred to have happened during wakefulness [Figure 1B, (13)]. Furthermore, non-fatal convulsive seizures infrequently result in a patient inverting into the prone position (39). It is generally agreed that ending a convulsive seizure in the prone position may contribute to SUDEP (39, 40). The most plausible explanation for this is that breathing during the postictal period is more likely to be impaired while prone consequent to upper airway occlusion or asphyxiation against the substrate on which the body is positioned (40–42).

The nose and mouth being pressed against pillows or the other accouterments of beds may impair postictal respiration by increasing inspiratory resistance and by causing the patient to rebreathe the trapped air (23, 43). Under normal circumstances, this obstruction of the airway would arouse the person and cause them to reposition, this response may not be possible in the time following a seizure (39, 44, 45). “Anti-suffocation” pillows are currently available; unfortunately, there is a paucity of evidence as to their effectiveness (23, 46).

Another sudden death condition, sudden infant death syndrome (SIDS) shares common features with SUDEP, including that patients are often found prone immediately following nighttime rest. SIDS rates were reduced significantly by the “Back to Sleep” campaign, which encourages care givers to place infants supine to sleep (47). A similar initiative has been proposed to reduce SUDEP rates (48); however, it is not clear whether sleeping in a supine position would be meaningfully protective against SUDEP as body position may change following a convulsive seizure (39).

## Sleep

Sleep alters respiratory, cardiac, and electrocerebral physiology in ways that may be relevant to SUDEP. During sleep, airway patency is decreased thereby increasing airway resistance and increasing the likelihood of airway occlusion (49, 50). Inspiratory drive is lower during non-rapid eye movement (NREM) sleep and lowest during rapid eye movement (REM) sleep (51). Chemical stimuli potently regulate breathing. Failure of the respiratory system to respond to rising CO_2_ and falling O_2_ levels consequent to seizure induced respiratory dysregulation is theorized to be important in SUDEP etiology. CO_2_ levels are higher during sleep (17, 51). The hypercapnic ventilatory response is attenuated in NREM sleep in comparison to wakefulness (51–55). The respiratory response to hypoxia is decreased in both NREM and REM sleep (56). Interestingly, the hypoxic ventilatory response of women is less affected by sleep than men (57). This difference in responsiveness to O_2_ depletion during sleep may be responsible for the decreased risk of definite, possible, and probable SUDEP in females (58, 59). Seizures which occur during sleep are associated with lower periictal blood oxygenation (60).

Cardiac and autonomic activity is also modulated by sleep in ways which are potentially relevant to SUDEP (61, 62). QT interval is longer during sleep than it is during wakefulness (15). It is hypothesized that a dramatic shift from parasympathetic to sympathetic drive may have a role in the dysregulation of cardiorespiratory function in SUDEP (63).

Sleep disordered breathing may also play a meaningful role in the nocturnality of SUDEP. Refractory epilepsy patients are at an increased risk of sleep disordered breathing, particularly obstructive sleep apnea [OSA; (64, 65)]. OSA increases a patient's risk of sudden cardiac death (66). Interestingly, sudden cardiac death in patients with obstructive sleep apnea happens more during the night which is similar to the temporal distribution of SUDEP but unlike that of sudden cardiac death in the general population which tends to happen more during the morning (66, 67). Obstructive sleep apnea is associated with autonomic dysfunction and lower resting oxygen saturation which might increase a patient's vulnerability to SUDEP (68–70). Whether OSA is increased in SUDEP cases has not been studied yet.

It is generally agreed that NREM sleep facilitates the occurrence of seizures and that seizures rarely occur during REM sleep (71, 72). Seizures which occur during sleep are longer and more likely to evolve into focal to bilateral tonic-clonic seizures (73). It is unclear whether the incidence of focal to bilateral tonic-clonic seizures among those with nocturnal seizures can be explained by the lower seizure threshold during sleep as this could also attributed to differences in epilepsy etiology (e.g., nocturnal seizures are more common in frontal lobe epilepsy) (74, 75). Regardless, the increased risk of a sleep-related seizure generalizing may confer an increased risk of SUDEP. Seizures which originate from sleep have more severe perturbation of cardiac activity (76). In an analysis of non-fatal seizures in patients who went on to die of SUDEP it was found that SUDEP victims had a larger surge in heart rate following seizures which happen during sleep in comparison to the seizures of patients who did not die of definite or probable SUDEP (63). It is not clear whether postictal generalized EEG suppression, a state which might facilitate SUDEP, is meaningfully altered by vigilance state of seizure origin. Some studies have observed that sleep increases the probability and duration of postictal generalized EEG suppression (60, 77–80). Conversely, other studies have not seen any association between sleep and postictal EEG suppression (81–83). In summary, there is some evidence to suggest that seizures which originate during sleep have different physiologic consequences than wake seizures in ways that are potentially meaningful to SUDEP.

The inherently unpredictable nature of SUDEP makes it difficult to study in humans; however, evoked seizures in animal models allow the physiological sequelae of seizures to be studied at any permutation of circadian phase and sleep state. Seizures which are induced during NREM sleep using maximal electroshock (MES) are longer, more severe, and more likely to result in death by seizure induced respiratory arrest than seizures induced during wakefulness (84). Non-fatal MES-induced seizures during NREM sleep also result in longer PGES, a greater degree of respiratory suppression, and longer apnea than seizures induced during wakefulness (84). Seizures induced in REM sleep in this model are universally fatal (84). The increased mortality seen after seizures induced during REM sleep is interesting given that seizures are less common during REM sleep (72, 85); however, this may not be true in some rodent models where REM sleep and the associated hippocampal theta rhythm might make seizures more likely (86). In one genetic mouse model with spontaneous seizures escalating sleep deficits preceded the fatal seizure suggesting that chronic sleep disturbances might play a role in SUDEP pathophysiology (87).

Because SUDEP is so frequently unobserved and rarely captured on EEG, it is not possible to determine the sleep state of origin for the fatal seizure in most cases. Patients who died of SUDEP are more likely to have had a nocturnal pattern of seizures and to have a history of seizures originating during sleep (12, 30, 63). As discussed above, meta-analyses of unwitnessed SUDEP cases classify a SUDEP case as being “sleep-related” if it happened at night and in the general vicinity of a bed. These criteria are suboptimal; notwithstanding, using these criteria, a majority of SUDEP cases are “sleep-related” [Figures 1A–D, (12, 13, 37)]. Due to the presence of EEG at the onset of the fatal seizure, the insights provided by the mortality in epilepsy monitoring units study (MORTEMUS) are crucial to teasing apart the role of sleep in SUDEP. In this study, seven of the 10 cases for which sleep state could be determined occurred during sleep (1 during REM, 1 during stage 1, 2 in stage 2, and 3 in sleep stages 3 or 4; Figure 1A, (10)).

## Circadian Rhythms

Circadian rhythmicity affects breathing independently of sleep state (17–20). Humans that are subjected to a constant routine paradigm, which spreads sleep and activity through the 24 h day, exhibit alterations in breathing at different times of day regardless of their sleep-wake state (16, 88, 89). Animal studies also demonstrate circadian differences in breathing (90, 91). Diurnal organisms, such as humans, are more active during the day and have greater ventilation during the day (88, 92). Conversely, nocturnal organisms such as rodents, which are more active during the night, display increased ventilation during the night (17, 91).

In humans, the hypercapnic ventilatory response is higher during the morning and afternoon but decreases substantially during the night (16, 88). There are also circadian differences in sensitivity to CO_2_ in rodents with a decrease in sensitivity during the day (90). The hypoxic ventilatory response is regulated in a circadian fashion in humans with greater sensitivity during the day (88, 92, 93); however, in rodents the response to hypoxic conditions is coupled to metabolism which changes in a circadian fashion in such a way that there are no net differences in the response to hypoxia at different times of day (91). The respiratory changes associated with seizures alter blood gas levels (94–97). Differences in how breathing responds to changes in blood gas levels at different times of day may alter a patient's ability to respond to seizure-induced respiratory changes. Lastly, respiratory tissues such as the larynx, trachea, and lung have peripheral circadian oscillators which operate under the purview of the central oscillator in the suprachiasmatic nuclei (SCN; 97). Bilateral SCN lesion disables the peripheral oscillators in these tissues as does genetic deletion of the clock genes cryptochrome 1 and 2 (98).

Circadian oscillations in baseline breathing, respiratory response to challenges, and clock gene expression in peripheral tissues are meaningful for a variety of disease states. The airway occlusion which is seen in sleep apnea is exacerbated by circadian changes in airway patency (99). Asthma is often worsened at night and respiratory irritants and allergens cause worse respiratory distress at this time (100–102). Chronic obstructive pulmonary disease symptoms are altered by circadian phase and these patients are more likely to require intubation in the morning (103). SIDS is thought to result, in part, from respiratory failure and occurs predominantly at night (104, 105).

The SCN is thought to play a role in autonomic regulation and thus explain why circadian changes may also impact cardiovascular control (106, 107); however, patients with impaired function of the SCN appeared to have similar cardiac function during sleep in comparison to healthy controls (108). Reduced heart rate variability (HRV) is an established risk factor for sudden cardiac death (109) and has been implicated in SUDEP risk although the few case-controls studies that have been published have conflicting findings (110–112). HRV is subject to circadian regulation in addition to the modulating effect of sleep state (113, 114). Day-night HRV dynamics appear to be altered in epilepsy patients; however, without identifying the role of sleep state or employing a forced desynchrony paradigm to isolate the influences of sleep state from circadian ones, it is difficult to state categorically whether this effect is mediated by sleep state or due to an independent circadian effect (115, 116). Cardiac responses to stimuli which are known to elicit a vagal response, such as compression of the eye, are regulated in a circadian fashion with the largest responses coming in late night and the early hours of the morning (117). QT lengthening or shortening may lower the threshold for ventricular fibrillation. Seizure-induced ventricular fibrillation may be seen in a minority of (near) SUDEP cases (118, 119). QT interval is modulated by both sleep state and circadian phase with QT intervals being longer during sleep and later in part of the night (15, 120).

It is well appreciated that seizures and interictal epileptiform discharges are regulated in a circadian manner (121–126). Analysis of seizure type, seizure timing, and sleep state of seizure origin indicates that sleep state and time-of-day independently affect seizures (124, 127). The influences of sleep state and circadian rhythms are also dependent on the site of seizure origin (128). It is unclear why the location of the seizure onset zone would alter the circadian distribution of seizures; however, it is known that different brain areas respond differently to the progression of circadian time (129).

Recently, infradian patterns in seizures have been identified which were previously underappreciated (130). These multidian rhythms have an influence on the occurrence of seizures which is comparable in strength to that of circadian phase. It is not clear whether seizures that happen at different points in these infradian oscillations are more likely to cause cardiorespiratory complications.

There are day-night differences in seizure severity and susceptibility consequent to DBA/2 audiogenic seizures and electrically induced seizures (131). It is postulated that these differences are causally related to day-night variations in serotonin and norepinephrine levels in different brain areas (131, 132). It is unclear on the basis of this study if there are any day-night differences in seizure induced death and whether these differences are independent of seizure severity. Furthermore, whether these differences are endogenously circadian, as opposed to being due to differential lighting conditions, was not investigated (131).

**Figure 2:**
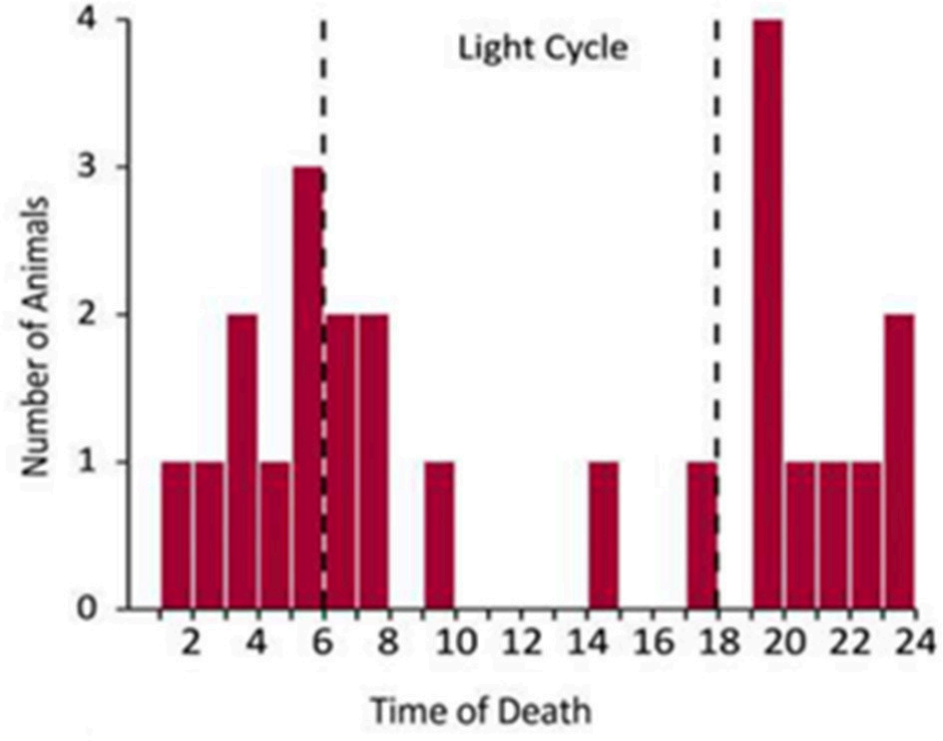
Temporal distribution of spontaneous seizure induced death in Kv1.1 knockout mice [redrawn with permission from Moore et al. (133)].

The only published data on the time-of-day of spontaneous death in an animal model of seizure induced death is from Kv1.1 null mice (133). These mice exhibit spontaneous seizures originating in the temporal lobe and typically die consequent to a seizure before 10 weeks of age. Kv1.1 null mice have an attenuated circadian rhythm in cardiac activity and the majority of their deaths occur during the night with peaks in mortality at the light/dark transition points [Figure 2, (133)]. This study did not monitor the vigilance state of the animal at the time of the fatal seizure, so it is impossible to determine whether this is an effect of sleep, circadian time, or both.

**Figure 3:**
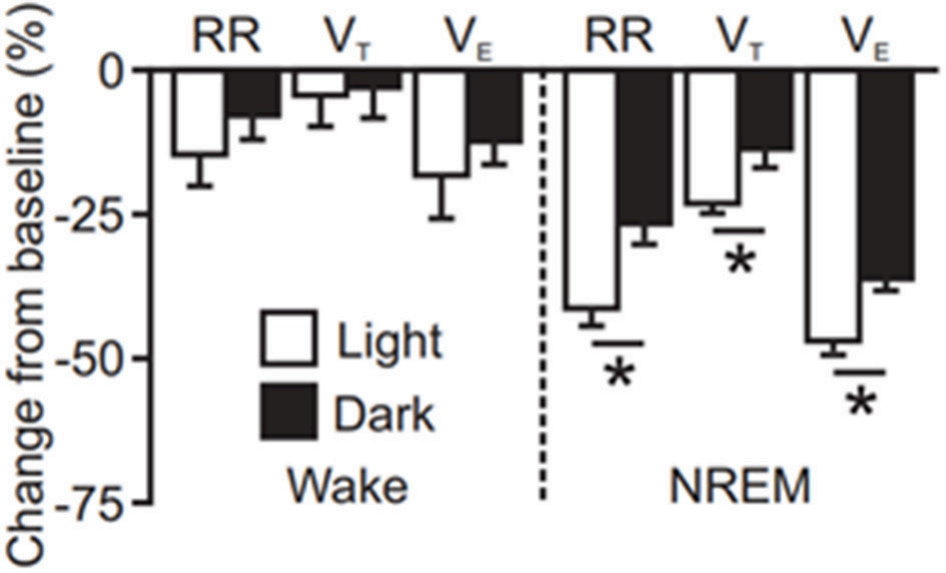
Quantification of seizure induced suppression of breathing (RR; respiratory rate, V_T_; tidal volume, V_E_; minute ventilation) following maximal electroshock seizures induced during wakefulness or non-rapid eye movement sleep (NREM) at different times of day [redrawn with permission from Purnell et al. (134)]. ^*^P < 0.05.

Seizures induced by MES during the day, the rodent inactive phase, are similar to seizures induced during the night in terms of duration and severity; however, MES seizures induced in the day during sleep resulted in a greater degree of postictal respiratory suppression (Figure 3, (134)). Seizures induced during this time also resulted in prolonged EEG suppression. This effect was even greater when seizures were also induced during sleep (134). Two caveats to this data are that only two time points were compared, and these experiments were conducted with the animals in a light-dark cycle environment. A broader sampling of time points throughout the 24 h day and conducting experiments in constant darkness, i.e., in the absence of circadian entraining light cues, may reveal a different temporal pattern.

SUDEP victims are more likely to have a nocturnal pattern of seizures (12, 14). Presently, it is impossible to determine whether this effect is driven by circadian rhythms or homeostatic sleep processes. Epilepsy surgery candidates, the population at the highest risk of SUDEP, have decreased HRV during the night compared to healthy controls (116). SUDEP is not uniformly distributed throughout the 24 h day (12). SUDEP presumed to have happened during sleep most commonly occurred between 0400 and 0800. SUDEP which is presumed to have happened during wakefulness most commonly occurred between the 0800 and 1200.The increased risk of seizures between 0400 and 1,200 is interesting as it suggests that there may be a circadian component to SUDEP which occurs independent of sleep state. If the nocturnality of SUDEP was attributable to sleep factors alone it would be expected that SUDEP frequency would decrease dramatically after 0800 when most people are no longer sleeping. In the MORTEMUS of SUDEP occurring in epilepsy monitoring units, most deaths occurred during the night. Conversely, most cases of near-SUDEP occurred during the day (10).

## Potential Mechanisms

### Serotonin

**Figure 4:**
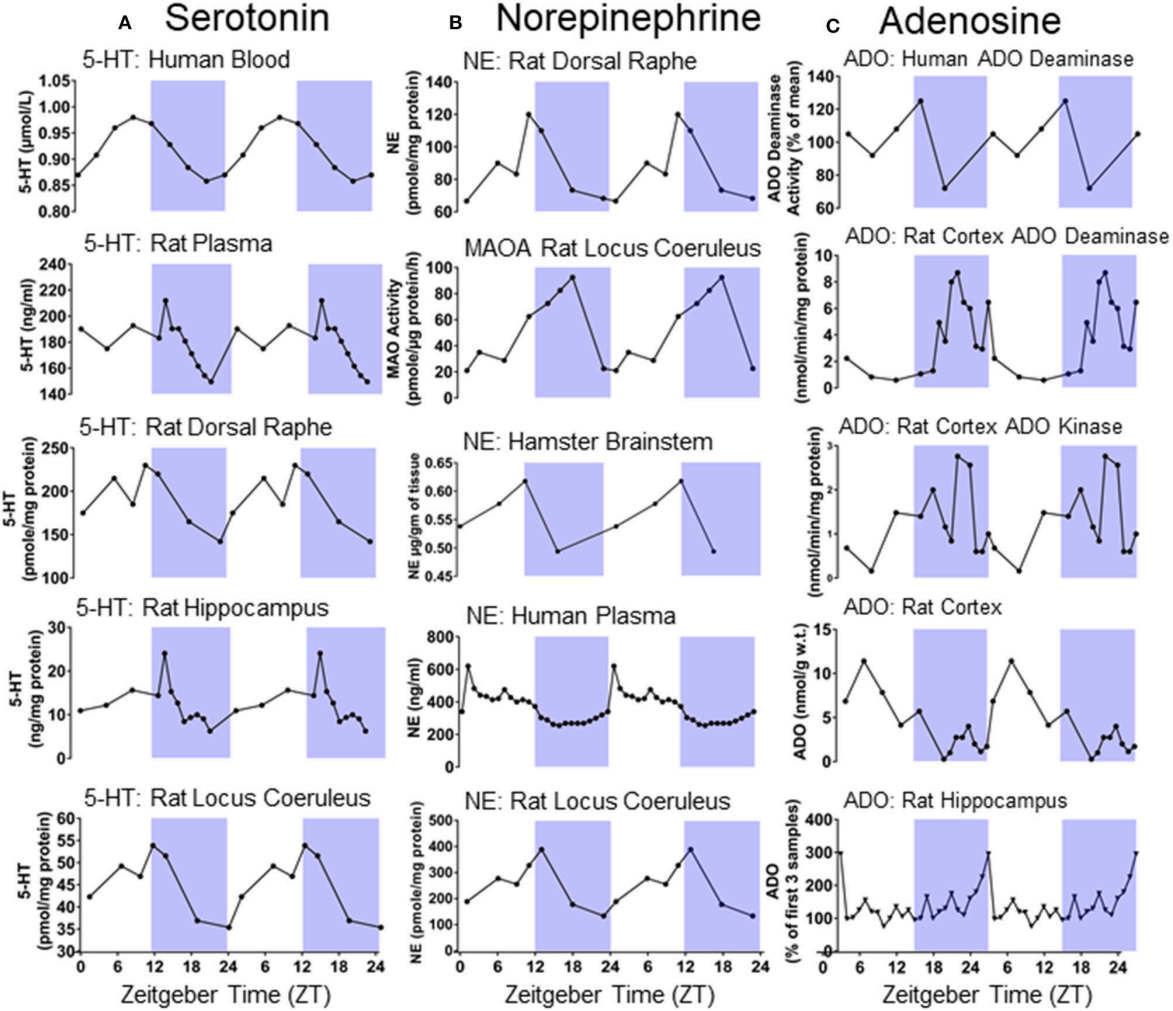
**(A)** Double plotted day/night differences in serotonin (5-HT) levels in different tissues of humans and rodents [redrawn with permission from Rao et al. (141); Mateos et al. (142); Agren et al. (132)]. **(B)** Double plotted day/night differences in norepinephrine (NE) and Monoamine oxidase A (MAOA) in different tissues of humans and rodents [redrawn with permission from (132, 143–145)]. **(C)** Double plotted day/night differences in adenosine (ADO) and its metabolizing agents in different tissues of humans and rodents [redrawn with permission from (146–148)].

In the central nervous system, serotonergic neurons are found in the raphe nuclei along the midline of the brainstem (135). Serotonergic neurotransmission modulates breathing, sleep-wake regulation, circadian rhythmicity, and seizures (136–139). Neuronal activity in the raphe nuclei is highest during wakefulness, reduced during NREM sleep, and almost entirely silent during REM sleep (140). Serotonin levels vary depending on circadian phase in areas such as the dorsal raphe, locus coeruleus, and hippocampus [Figure 4, (132, 141, 142, 149)]. Seizures suppress serotonergic neurotransmission in the ictal and the postictal period (150). Increases in serotonergic neurotransmission is a critical component of the arousal response to inspired CO_2_ (151–153). Stable breathing requires serotonergic neurotransmission (138). Seizure induced disruption of normal serotonergic arousal mechanisms may prevent the normal arousal response to CO_2_ in the postictal period and facilitate death (105).

In epilepsy patients, selective serotonin reuptake inhibitors (SSRI) reduce seizure associated hypoxemia (95). Larger seizure induced changes in serum serotonin are associated with a reduction in the tonic phase of a convulsive seizure (154). Furthermore, interictal serum serotonin levels are associated with shorter PGES (154). In the DBA/1 mouse model of seizure induced respiratory arrest, administration of the SSRIs fluoxetine, fluvoxamine, paroxetine, sertraline, and fluoxetine prevent respiratory arrest and death (155–158). The likelihood of seizure induced respiratory arrest is also reduced by administration of 5-hydroxytryptophan, a molecule required in serotonin synthesis (159). Conversely, serotonin antagonism increases the likelihood of seizure induced respiratory arrest consequent to audiogenic seizures (157). Seizures are more severe and more likely to be fatal in mice with a genetic deletion of serotonin neurons in the central nervous system (*Lmx1b*^*f*/*f*/*p*^) mice vs. wild type counterparts. Seizure induced death is reduced following MES by SSRIs and 5-HT_2A_ receptor agonists, but not a 5-HT_2C_ agonist (160). Times in which serotonergic activity is lower, such as the during the night, may lower seizure threshold and make seizures which do occur more dangerous (149, 161, 162)

### Adenosine

Adenosine is a purinergic transmitter which is found in many brain areas and known for its role in sleep-wake regulation, breathing, epilepsy, and a variety of other diseases (163–166). Adenosine accumulation and clearance is regulated in a circadian fashion in a variety of brain areas [Figure 4, (146–148, 167)]. Adenosine levels increase during wakefulness and are depleted during sleep (163, 168). The sleep disturbances often associated with epilepsy may be explained by alterations in adenosine signaling (169). Adenosine is an endogenous anticonvulsant and adenosine levels increase during seizures (170, 171). Furthermore, manipulations to adenosine or its clearance modulate epileptogenesis (171–173). Having recurrent seizures may, in turn, decrease, or increase adenosine levels in different brain areas (169). Adenosine analogs applied to the brainstem of rats cause prolonged suppression of breathing (174, 175). Adenosine analogs administered intracerebroventricularly decrease respiration and elicits apnea in cats (176). Inhibition of adenosine clearance initially prevents the escalating severity of motor seizures following kainate injection; however, the adenosine kinase inhibited animals quickly progress to more severe motor seizures and invariably die, whereas animals not subjected to adenosine kinase inhibition do not die. Treating with caffeine following seizure onset prolongs survival in mice subjected to inhibition of adenosine clearance prior to seizure induction with kainate (177). These results suggest that an unchecked surge in adenosine consequent to a seizure may result in precariously increased levels of neuronal inhibition and thereby facilitate death (177).

### Norepinephrine

Norepinephrine, a catecholaminergic neurotransmitter found in the rostral brainstem including in the locus coeruleus, modulates seizure activity, (178) breathing, (179), and is subject to circadian regulation in an array of different brain areas [Figure 4, (132, 143–145)]. Like serotonin, norepinephrine promotes wakefulness and is an important part of the ascending arousal system (180). In DBA/1 mice, the selective norepinephrine reuptake inhibitor venlafaxine and the SSRIs fluoxetine and fluvoxamine, which also potentiate noradrenergic activity, are more effective in preventing seizure induced respiratory arrest than the selective SSRI paroxetine (155, 157). Respiratory arrest is also reduced in DBA/1 mice with the norepinephrine reuptake inhibitor atomoxetine (181, 182). In light of this evidence, times at which noradrenergic tone is low might make seizure induced respiratory arrest more likely.

## Summary

**Figure 5:**
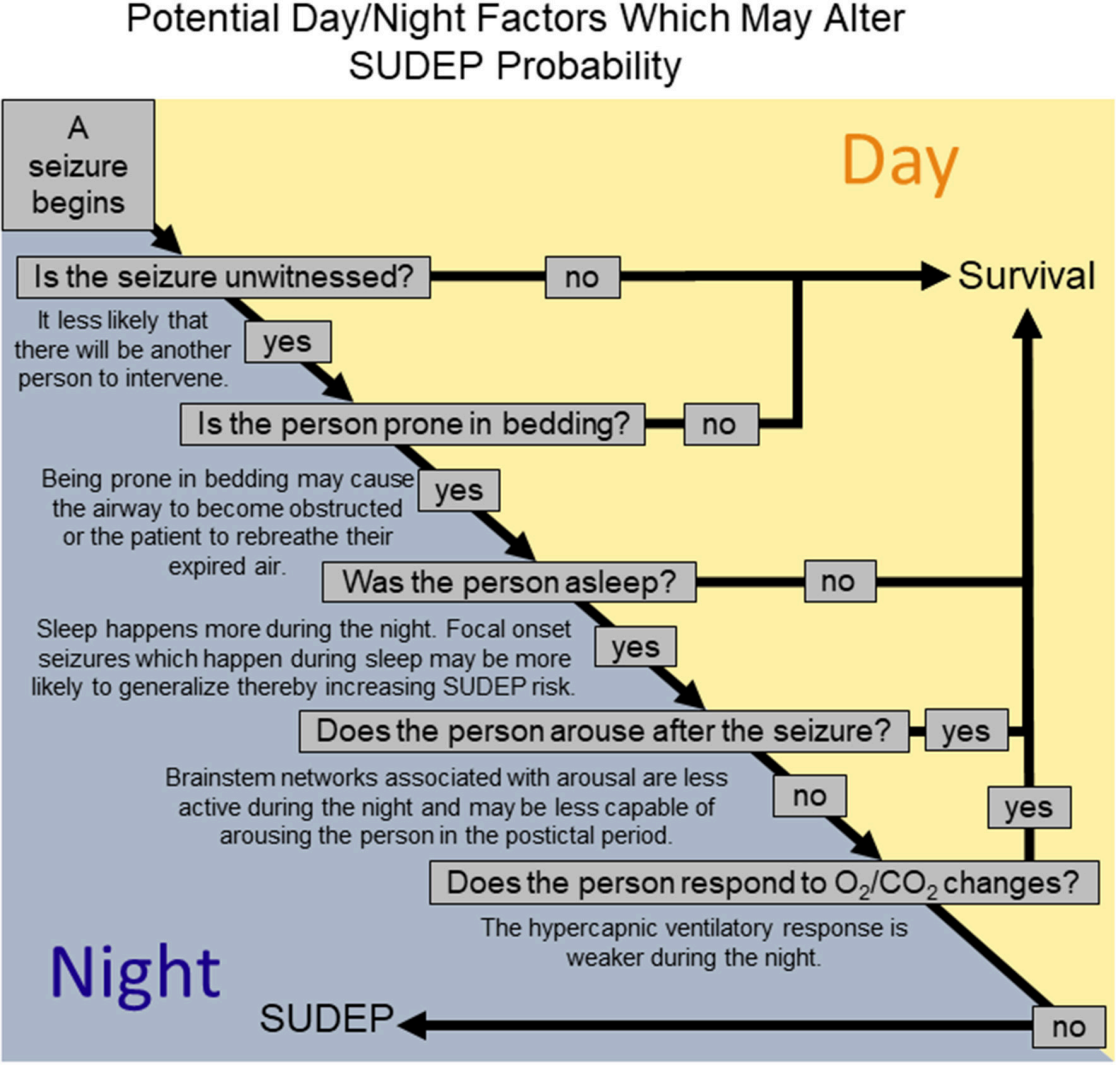
A schematic representation of how different factors relevant to the night might alter the likelihood that a seizure results in SUDEP.

The reason that SUDEP happens more during the night is likely multifactorial involving both situational factors, such as being unattended, and physiological changes due to the influence of sleep and circadian rhythms. Human studies suggest that being without a witness and prone following a seizure, which is more likely during the night, might increase risk for nocturnal SUDEP. At the same time, experimentation in animal models and observation of human seizures indicate that both sleep and circadian phase may adversely affect postictal cardiovascular recovery. Sleep and circadian phase have additive effects on breathing which may compound in some way to produce a hazardous postictal state. Similarly, it may be that sleep, and circadian phase have additive effects on vulnerability to seizure induced respiratory arrest. When the factors associated with being without a witness and prone are added to the mix along with the potential effects of sleep and circadian phase SUDEP might be more likely (Figure 5).

Altering the circumstances in which a seizure occurs is currently the best way for reducing the probability of nocturnal SUDEP, but it is not enough. Patients who do not sleep alone or are being monitored by the use of a device seem to be somewhat protected against SUDEP; however, numerous SUDEP cases have occurred in the direct presence of medical professionals and none of their interventions were sufficient to prevent death. Families and caregivers should be educated about SUDEP and given instruction in basic seizure first aid; however, it should be made abundantly clear that such interventions might be sufficient to prevent death, but it might not and those who have lost someone due to SUDEP are in no way at fault. The risk of SUDEP, nocturnal and otherwise, should be taken into account by patients considering any choice which might alter their likelihood of having a seizure such as adherence, titrating off their medications, switching medication, or pursuing surgical interventions or other non-pharmacological measures.

## Author Contributions

BP drafted the initial document which was edited by RT, GB, and BP.

### Conflict of Interest Statement

The authors declare that the research was conducted in the absence of any commercial or financial relationships that could be construed as a potential conflict of interest.
